# Novel CuO/Mn_3_O_4_/ZnO nanocomposite with superior photocatalytic activity for removal of Rabeprazole from water

**DOI:** 10.1038/s41598-021-94066-y

**Published:** 2021-07-26

**Authors:** Sauvik Raha, Dipyaman Mohanta, Md. Ahmaruzzaman

**Affiliations:** Department of Chemistry, National Institute of Technology Silchar, Silchar, Assam 788010 India

**Keywords:** Nanoscale materials, Pollution remediation

## Abstract

In this work, a nanohybrid of CuO/Mn_3_O_4_/ZnO was generated through a simple hydrothermal based procedure. The CuO/Mn_3_O_4_/ZnO nanohybrid has been characterized using X-ray diffraction, transmission electron microscopy high resolution transmission electron microscopy, X-ray photoelectron spectroscopy, scanning electron microscopy and energy dispersive X-ray analysis. UV–visible spectrophotometry and photoluminescence techniques allowed evaluation of optical properties that additionally suggested the prevalence of strong interfacial interaction between the three moieties of the nanohybrid and suppressed electron–hole recombination. The hybrid photocatalyst brought on ~ 97.02 ± 1.15% disintegration of rabeprazole when illuminated with visible light. The progress of the photodegradation was in conformity with pseudo-first order kinetic model and had a velocity constant of 0.07773 min^−1^. Additionally, ~ 84.45% of total organic carbon removal was achieved while chemical oxygen demand was reduced by ~ 73.01%. Using high resolution liquid chromatograph mass spectrometry technique, identification of the degraded products was made and accordingly the mechanistic route of the aforesaid degradation was proposed.

## Introduction

There has been a great rise in the consumption of pharmaceutical drug over the last few decades and consequentially their occurrence in aquatic regions and drinking water supplies have also spiraled up without precedence. The presence of such biologically non-degradable organic contaminants has increasingly posed threat to the aquatic fauna and flora as well as terrestrial organisms as they carry the potential to cause severe disruption of all sorts of biological activities. Even human beings have failed to escape the adverse effects generated from the presence of such xenobiotic substances in the ecosystem^1,2^.

Among many other emerging organic water pollutants, pharmaceutical waste is of great concern^3,4^. Pharmaceutical substances often get into water bodies by human excretion and their inappropriate disposal of hospital waste^5,6^. Because of the ability to interact with living bodies, pharmaceutical wastes often impose threat to the aquatic ecosystem^7^. The increasing accumulation of pharmaceuticals marine biomes directly or indirectly affects the flora and fauna and in turn disturbs the ecological balance. Rabeprazole is a pharmaceutical drug that is widely recognized for its acid-inhibiting ability and is commonly employed for the treatment of a number of acid-related disorders^8–11^. The excessive use and indiscriminate disposal of rabeprazole lead to their accumulation in water bodies. Nevertheless, the drug can cause several side-effects that include headache, abdominal pain, constipation, dizziness, cramps, spasms and a great deal more^12,13^. The presence of even trace amount of rabeprazole in water bodies can wreak untold havoc on all forms of life in the aquatic ecosystem. Therefore, efforts have been made to rid water of such organic contaminants. In this regard, the fabrication of an efficient nano-scaled photocatalyst has drawn much attention^14,15^. This is owing to the fact that in comparison with other wastewater treatment techniques, photocatalytic degradation involves not just removal but complete breakdown of otherwise recalcitrant organic contaminants sans the generation of any harmful products^16–20^. This has led to the advent of a new set of oxidative procedures in the field of photocatalysis called Advanced Oxidation Process (AOP) aimed at total breakdown of persistent organic contaminant such as rabeprazole through generation of reactive oxygen species^21–24^.

Heterogenous photocatalysis is a class of AOP that has proved effective in the removal of persistent organic contaminants from aquatic environment^25^. Among metal oxide semiconductors, ZnO with a band gap of ~ 3.37 eV, superior electrochemical stability, high electron mobility, favourable isoelectric point of ~ 9 and promising electro-optical properties has been widely used in fabricating visible light active coupled photocatalyst systems^26^. Although alone, wide band gap limits its applicability within the ultra-violet spectrum of irradiation^25,26^. Also, as a photocatalyst, its performance has been found to be afflicted by intense charge recombination. Sustaining a wide separation of photo-generated charge carriers would require fine tuning the band gap of ZnO by coupling with other narrow band gap semiconductor metal oxides with compatible band edges. The band gap of pristine ZnO is ~ 3.37 eV. Pristine ZnO nanoparticles do not harvest visible LED light efficiently as incident radiation does not have sufficient energy to generate electron–hole pairs. However, n–n or n-p nanoheterojunction with narrow band gap semiconductors such as ZnO/CdS^27,28^, ZnO-MnO_2_^29,30^, ZnO/CuO^31,32^ have reported to have synergistically enhanced the charge separation. Moreover, the coupling of narrow band gap material also enhances visible light harvesting capacity of the catalyst, thereby, increasing the efficiency under LED irradiation. Recently, ternary metal oxide nanocomposite such as ZnO/MnO_2_/Cu_2_O^33^, ZnO/Fe_2_O_3_/MnO_2_^34^, ZnO/MnO_2_/Gd_2_O_3_^35^ have been thoroughly investigated for synergistically enhanced photocatalytic performance under visible light irradiation condition. The ternary nanoheterojunction formation with compatible valence band-conduction band position can greatly augment the photocatalytic properties of metal oxide nanoparticles. The authors of the current aimed at an economically viable tailoring of the band edges of ZnO by coupling ZnO with low cost CuO and Mn_3_O_4_, both of which having narrow band gaps and compatible band structures, are excellent candidates for harvesting visible light. CuO usually has a band gap of ~ 1.70 eV and in coupled systems has demonstrated remarkable photocatalytic capacity^36,37^. Mn_3_O_4_ with a band gap of ~ 2.10 eV could further reinforce separation of photogenerated charge carriers. Of late, Mn_3_O_4_ based photocatalysts have exhibited superlative response to visible light driven photocatalysis^38,39^.

In this work, a facile fabrication technique of CuO/Mn_3_O_4_/ZnO multicomponent photocatalyst architecture based on hydrothermal route has been presented and a detailed investigation has been undertaken to check the photocatalytic efficiency of the nanohybrid vis-à-vis degradation of a prototype recalcitrant organic contaminant, rabeprazole in its aqueous phase. The photocatalytic degradation over the ternary nanohybrid was accomplished by 60 min and a degradation efficiency of 97.02 ± 1.15% was attained. The photodegradation reaction was found to be consistent with pseudo-first order kinetic model with a velocity constant of 0.07773 min^−1^. The influences of co-existing substances and real water samples on photocatalytic degradation of the target organic contaminant was also briefly studied in order to gain better sight vis-à-vis applications in real environment. The fabrication of a ternary heterojunction of CuO/Mn_3_O_4_/ZnO and its application in the elimination of rabeprazole with a study about the interactive influences of various co-occurring species and water samples mark the novelty of this work.

## Experimental section

### Materials and measurement

AR grade reagents such as zinc nitrate hexahydrate (> 98%), manganese chloride tetrahydrate (> 98%), copper acetate monohydrate (> 98%), sodium hydroxide (> 98%), deionized water etc. were purchased from Sigma Aldrich and were used without additional purification.

### Synthesis

#### Synthesis of the nano-scaled materials

To aqueous solution of 25 mmol of copper acetate monohydrate, 1 mL of glacial acetic acid was added and the solution was heated to 100 °C. This was followed by drop-wise addition of an aqueous solution of NaOH under magnetic agitation. The solution color gradually changed from blue to black, and a considerable amount of black precipitate (CuO) was formed^40^. To this was added 25 mmol of zinc nitrate hexahydrate (Zn(NO_3_)_2_.6H_2_O) followed by addition of drops of aqueous solution of NaOH. The reaction mixture was magnetically agitated and then to it was added 25 mmol of manganese chloride tetrahydrate (MnCl_2_. 4H_2_O) followed by a dropwise addition of an aqueous NaOH solution. After thorough magnetic agitation of this reaction mixture, it was next shifted to an autoclave. The autoclave was then put inside an oven maintained at 100 °C. The reaction mixture was kept in this condition for 24 h. The dark brown residue thus formed was collected, rinsed repeatedly with ethanol and then dried. The dried sample was calcined at 300 °C in a muffle furnace for 4 h. The calcined sample was given the tag CuO/Mn_3_O_4_/ZnO. Other samples of Mn_3_O_4_/ZnO, CuO/ZnO, CuO/Mn_3_O_4_, ZnO, CuO and Mn_3_O_4_ were prepared with the relevant starting materials following the same aforementioned procedure.

#### Characterization

For evaluation of the crystalline structure of CuO/Mn_3_O_4_/ZnO, Mn_3_O_4_/ZnO, CuO/ZnO, CuO/Mn_3_O_4_, ZnO, Mn_3_O_4_ and CuO, all the samples were analyzed by Bruker D8 Advance X-ray diffractometer with Cu-K_α_ radiation. JEOL JEM 2100 instrument was used to carry out TEM and SAED analyses of CuO/Mn_3_O_4_/ZnO. PHI 5000 Versa Prob II spectrometer was used to conduct X-ray photoelectron spectroscopy of CuO/Mn_3_O_4_/ZnO. SEM micrographs and EDAX spectrum were obtained for the final nanocomposite using JEOL Model JSM—6390LV. HRLCMS was done with 1290 Infinity UHPLC System, Agilent Technologies, USA. TOC analyses were conducted with Elementar, Liqui TOC. Photoluminiscence data of CuO/Mn_3_O_4_/ZnO, Mn_3_O_4_/ZnO, CuO/ZnO, CuO/Mn_3_O_4_, ZnO, Mn_3_O_4_ and CuO were obtained with Hitachi F4600 equipment. For recording the absorbance spectra of the samples, GENESYS 10S UV–visible spectrophotometer was used. Photocurrent (PC) measurements were carried out on a Biologic SP-200 electrochemical workstation with a standard three-electrode cell at room temperature. Electrochemical impedance spectroscopy (ESI) was performed using a potentiostat with a sinusoidal perturbation voltage of 2 mV and the frequency range of 0.01 Hz to 1 MHz. The concentration of leached metal ions was determined using a Hitachi 180–70 atomic absorption spectrometer.

#### Evaluation of photocatalytic activity

The photocatalytic activity of the designed nano-hybrid photocatalyst was put to evaluation by monitoring its ability to cause disintegration of rabeprazole in its aqueous medium underneath an LED illumination and the whole experiment was executed in a chamber that had in it a Philips white LED bulb of 23 W. A luxmeter was used to measure lux and radiation intensity. Illuminance measured 11,830 lx and radiation intensity registered 48.25 W m^−2^. The degradation reaction was executed at room temperature. The drug solution alongside the photocatalyst was mechanically agitated for a span of 30 min in order to attain adsorption–desorption equilibrium. Thereafter, the absorbance was recorded. Next, the reaction mixture was subjected to LED irradiation for the attainment of photocatalytic degradation. The photodegradation was monitored by tracking the maximum absorbance of rabeprazole at 290 nm at an interval of 10 min for a total span of 60 min.

The following equation was employed to evaluate the efficiency of degradation:

1 $$Degradation\, efficiency \left(\%\right)=\left(\frac{{C}_{0}-C}{{C}_{0}}\right)\times 100$$

where C_0_ and C stand for the respective concentrations of rabeprazole at t = 0 and t = t.

For the assessment of kinetics of the photodegradation the equation underneath was used:

2 $$ln\frac{{C}_{0}}{C}=kt$$

where C_0_ and C designate rabeprazole concentrations at t = 0 and t = t and k is the velocity constant of the pseudo-first order reaction in min^−1^. The error bars in the diagrams displaying photodegradation represent minor standard deviations and are indicative of remarkable reproducibility over five repetitions of the experiment under a given set of conditions.

Trapping experiments using various scavengers were carried out for ascertaining the involvement of reactive species in the photodegradation of rabeprazole. Furthermore, the photodegradation capacities were also evaluated in terms of parameters such as chemical oxygen demand and total organic carbon.

## Results and discussion

### XRD studies

**Figure 1:**
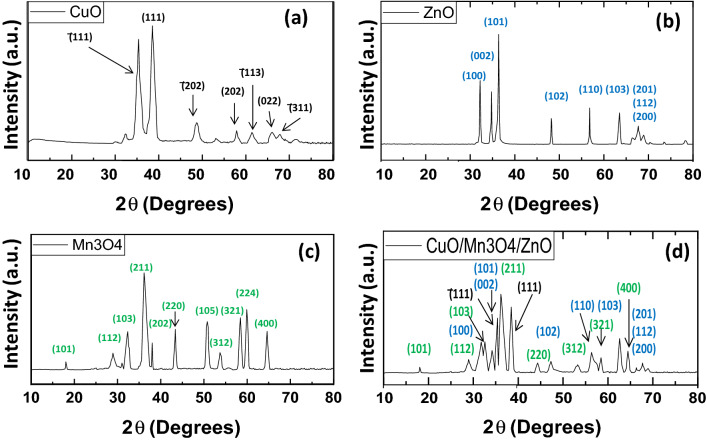
XRD patterns of pristine CuO, pristine ZnO, Pristine Mn_3_O_4_ and ternary CuO/Mn_3_O_4_/ZnO nanocomposite.

XRD analysis was done for determination of the crystalline phases of the compounds prepared. Figure 1(a–d) shows the XRD patterns of pristine CuO, pristine ZnO, Pristine Mn_3_O_4_ and the ternary nanocomposite CuO/Mn_3_O_4_/ZnO. The XRD pattern (Fig. 1a) of the synthesized copper oxide matched with the previously recorded XRD pattern of CuO registered as JCPDS 89-5899. Peaks at 2θ values of 35.38°, 38.58°, 48.71°, 58.06°, 61.41°, 66.02° and 66.42° could be associated with ($\overline{1}11$), (111), ($\overline{2}02$), (202), ($\overline{1}13$), (022) and ($\overline{3}11$) planes respectively. The XRD pattern of pristine zinc oxide (Fig. 1b) showed peaks in its diffraction pattern at 2θ values of 31.65°, 34.33°, 36.25°, 47.43°, 56.41°, 62.68°, 66.23°, 67.81° and 68.98° that could be associated with (100), (002), (101), (102), (110), (103), (200), (112) and (201) of ZnO phase (JCPDS 89-1397). Also, the XRD pattern of pristine Mn_3_O_4_ (Fig. 1c) sample displayed peaks 18.01°, 28.92°, 32.39°, 36.31°, 44.47°, 53.83°, 58.55° and 64.64° that could be identified as diffracted from (101), (112), (103), (211), (220), (312), (321) and (400) planes of Mn_3_O_4_ phase (JCPDS 89–4837).

The XRD data of the CuO/Mn_3_O_4_/ZnO ternary nanocomposite exhibited peaks of ZnO, Mn_3_O_4_ and CuO phases and labeled in Fig. 1(d). Diffraction from planes (100), (002), (101), (102), (110), (103), (200), (112) and (201) of ZnO phase (JCPDS 89-1397) gave rise to peaks positioned at 31.61°, 34.30°, 36.21°, 47.41°, 56.37°, 62.63°, 66.20°, 67.78° and 68.94° respectively. Peaks at 2θ values of 35.36° and 38.54° could be associated with (®111) and (111) crystallographic planes of CuO (JCPDS 89–5899), respectively. The remaining peaks at 18.01°, 28.84°, 32.28°, 36.21°, 44.35°, 53.76°, 58.46° and 64.54° could be respectively associated with (101), (112), (103), (211), (220), (312), (321) and (400) planes of Mn_3_O_4_ phase (JCPDS 89–4837).

The average crystallite size of the synthesized nanoparticles were calculated by using the Debye–Scherrer’s equation, $D=\frac{k\lambda }{\beta cos\theta }$ where D is the crystallite size in nanometers, k is the shape factor (0.89), λ is the wavelength of *CuK*α radiation (λ = 1.54056 Å), β is full width at half maximum (FWHM) of the particular peak and θ is the Bragg’s angle. The average particle sizes of pristine CuO, pristine ZnO, Pristine Mn_3_O_4_ and CuO/Mn_3_O_4_/ZnO ternary nanocomposite were found to be approximately 10 nm, 20 nm, 14 nm and 8 nm respectively.

### TEM and SAED analyses

**Figure 2:**
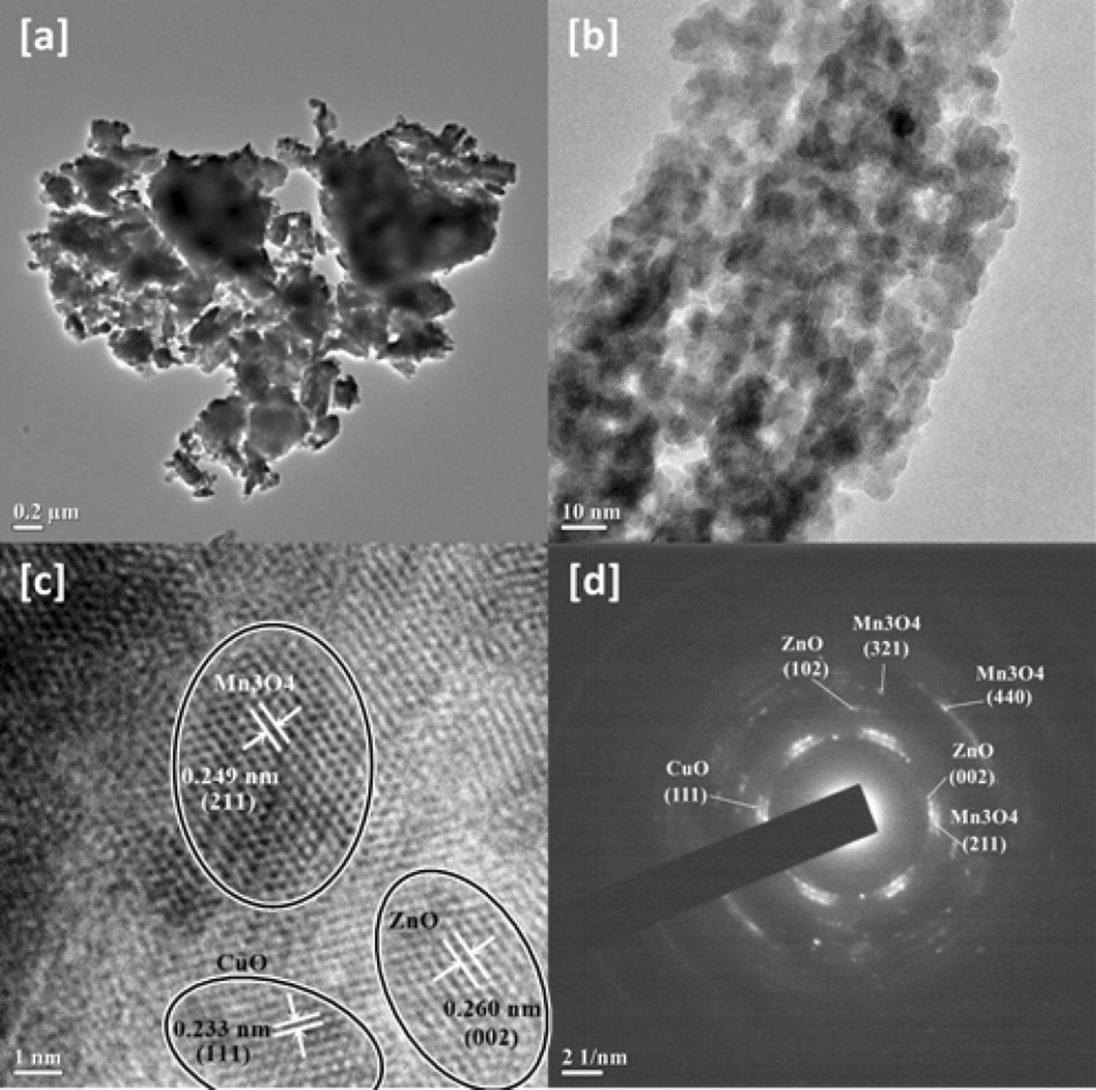
(**a**) TEM micrograph, (**b**,**c**) HRTEM micrographs and (**d**) SAED patterns of CuO/Mn_3_O_4_/ZnO.

Transmission Electron Spectroscopy (TEM) was employed to analyze the morphology of the engineered nanohybrid and gain further insights of its structure. The TEM micrograph (Fig. 2a) revealed images of scattered nanoparticles of ZnO, Mn_3_O_4_ and CuO in contact with each other and in all likelihood interfacial heterojunctions have been formed by them. From the separation of lattice fringes viewable in the micrographs of High- Resolution Transmission Electron Spectroscopy (HRTEM) (Fig. 2b,c), the particles could be identified. The three distinguished lattice fringes with inter-planar spacings of 0.260 nm, 0.249 nm and 0.233 nm could be associated with the (002) facet of ZnO phase (JCPDS 89-1397), (211) crystallographic plane of Mn_3_O_4_ phase (JCPDS 89-4837)^38^ and (111) facet of CuO (JCPDS 89-5899)^41^. The average particle size was measured to be 8.34 nm. The SAED patterns (Fig. 2d) had concentric rings which were indicative of the polycrystalline nature of the sample. The lattice planes of the three phases were duly identified and marked.

### XPS studies

**Figure 3:**
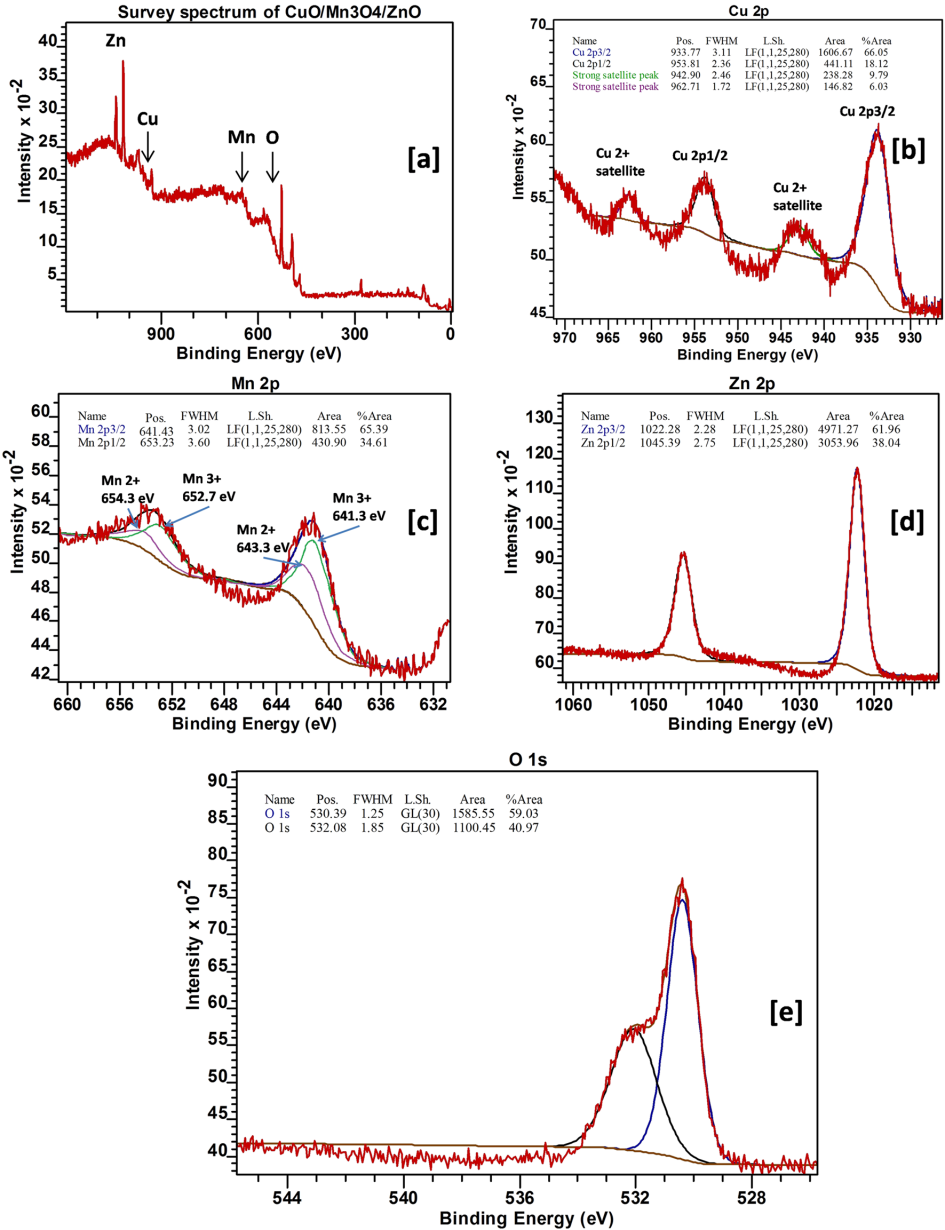
(**a**) XPS survey spectrum of CuO/Mn_3_O_4_/ZnO and HR-XPS spectra of (**b**) Cu, (**c**) Mn, (**d**) Zn and (**e**) O in the final nanocomposite.

The XPS survey spectrum (Fig. 3a) of CuO/Mn_3_O_4_/ZnO revealed peaks that could be associated with Zn, Mn, Cu and O. The photoelectron peaks observed in the core level spectrum of Cu 2p (Fig. 3b) at ~ 933.77 eV and ~ 953.81 eV corresponded to Cu 2p_3/2_ and Cu 2p_1/2_ peaks for Cu. The presence of two strong satellite peaks at ~ 938.62 eV and ~ 958.40 eV additionally confirmed the presence of Cu (II) in the sample^42,43^. The HR-XPS spectrum of Mn 2p (Fig. 3c) also exhibits two peaks due to spin orbit splitting at ~ 641.43 eV and ~ 653.23 eV that could ascribed to Mn 2p_3/2_ and Mn 2p_1/2_ electronic states of Mn_3_O_4_. The energy difference of 11.80 eV between them further confirmed the formation of Mn_3_O_4_ phase in the nanocomposite^44^. The Mn 2p_3/2_ and Mn 2p_1/2_ peaks were deconvoluted into four peaks centered at ~ 641.3 eV, 652.7 eV, ~ 643.3 eV and 654.3 eV which were attributed to Mn^3+^ 2p_3/2,_ Mn^3+^ 2p_1/2_ , Mn^2+^ 2p_3/2_ and Mn^2+^ 2p_1/2_ respectively. Moreover, the ratio of peak area under the curve of Mn^2+^ to Mn^3+^ was found to be 1:2 which indicated successful fabrication of Mn_3_O_4_^45^. The photoelectron peaks (Fig. 3d) at ~ 1022.28 eV and ~ 1045.39 eV of the Zn 2p core level spectrum could be indexed to Zn 2p_3/2_ and Zn 2p_1/2_ peaks for Zn^2+^^46^. The peak (Fig. 3e) corresponding to ~ 530.39 eV corresponded to O 1 s of O^2−^ lattice array. And the other peak at around ~ 532.08 eV in all probability could be due to various oxygen defects and surface adsorbed oxygen species^47,48^. The slight shifts observed in these binding energy values than those previously reported indicated interfacial interactions between the individual metal oxides and therefore a further confirmation of the successful fabrication of the intended coupled photocatalyst system. A likely redistribution of different electron densities in individual metal oxides of the nanocomposite due to strong interaction at interfaces might account for these binding energy shifts^49^.

### SEM and EDAX studies

**Figure 4:**
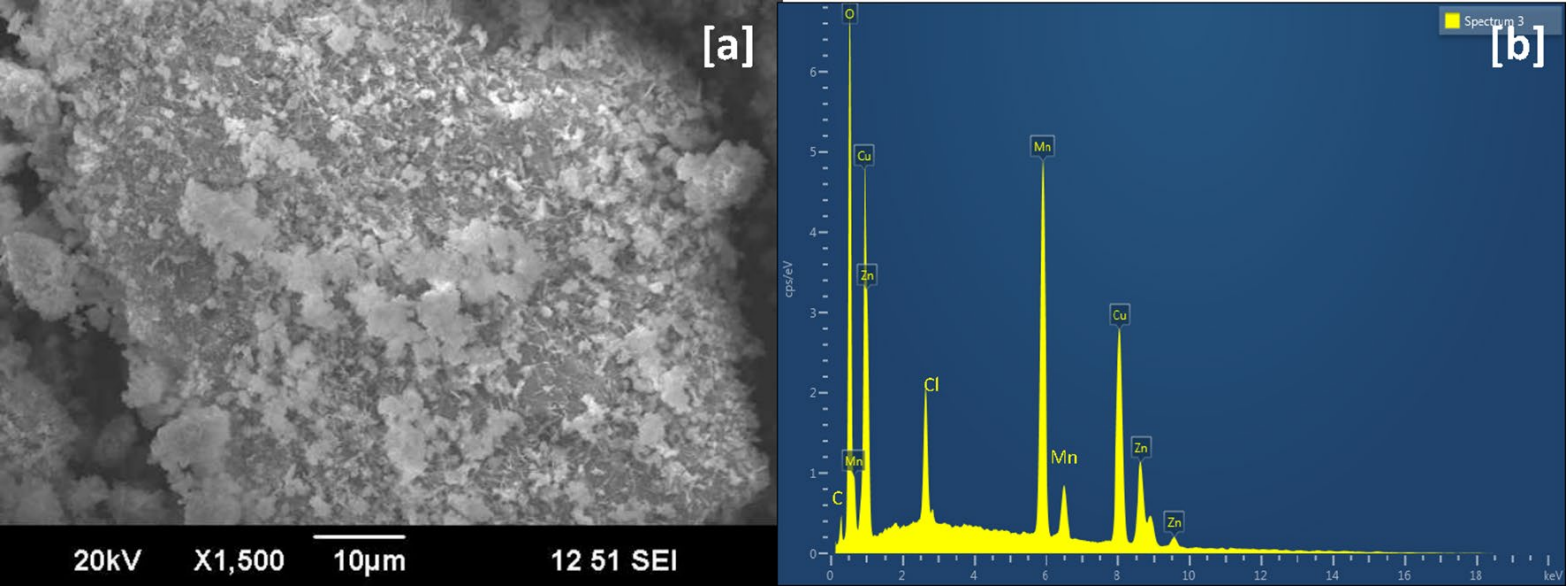
(**a**) SEM micrograph and (**b**) EDAX spectrum of CuO/Mn_3_O_4_/ZnO.

SEM image (Fig. 4a) of the CuO/Mn_3_O_4_/ZnO revealed the surface morphology of the nanocomposite. Agglomerated clusters of nanomaterials can be observed with high porosity. EDAX of CuO/Mn_3_O_4_/ZnO nanocomposite (Fig. 4b) showed signals corresponding to Zn, Mn, Cu and O in the spectrum. The peaks at around ~ 8.6 keV, ~ 5.9 keV, ~ 8.0 keV and ~ 0.5 keV could be respectively associated with Zn, Mn, Cu and O, all corresponding to the K-series emissions. The L-series emissions of Zn, Mn and Cu were also observed at ~ 1.01 keV, ~ 0.93 keV and ~ 0.64 keV respectively. The atomic percentages of these elements given in Table 1 are suggestive of the successful synthesis of CuO/Mn_3_O_4_/ZnO nanostructures with substantial physical integration among the individual components. The atomic percentages further suggest the presence of CuO, Mn_3_O_4_ and ZnO in the ratio ~ 1:1:1 in the nanohybrid. Further the absence of impurity peaks in EDAX spectrum was fairly consistent with the neat XRD plot obtained for the ternary nanocomposite.

**Table 1:** Elemental composition of the final nanocomposite CuO/Mn_3_O_4_/ZnO from EDAX spectrum.

Element	Line type	Weight%	Atomic%
O	K-series	24.62	54.50
Cu	K-series	16.28	9.09
Mn	K-series	42.31	27.27
Zn	K-series	16.79	9.14
Total		100	100

### Optical properties

**Figure 5:**
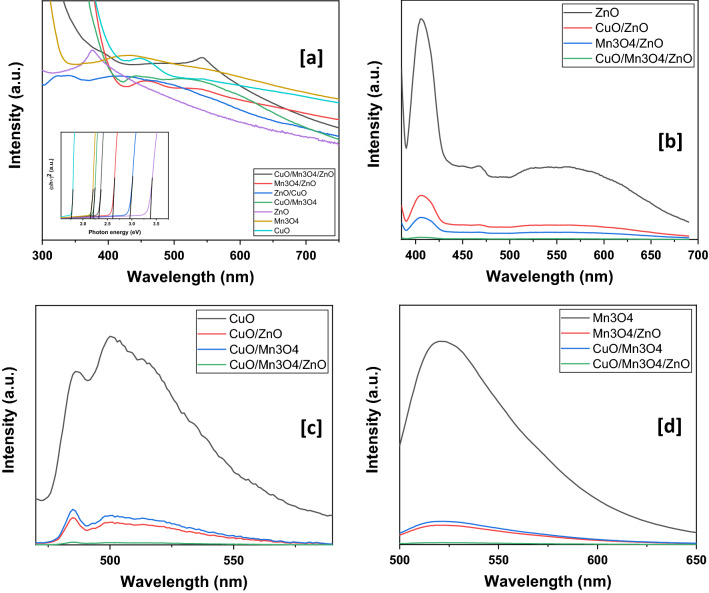
(**a**) UV–visible absorbance spectra of the various nano-scaled samples with an inset showing their respective Tauc’s plot for calculation of band gaps and (**b**–**d**) photoluminiscence graphs.

The UV–visible spectra (Fig. 5a) of CuO/Mn_3_O_4_/ZnO, Mn_3_O_4_/ZnO, CuO/ZnO, CuO/Mn_3_O_4_, ZnO, Mn_3_O_4_ and CuO were recorded to assess their optical properties. Pristine ZnO was found to have a maximum absorbance at ~ 375 nm. Mn_3_O_4_ absorbance spectrum demonstrated a hump centered about ~ 430 nm while pristine CuO had a maximum absorbance at ~ 449 nm. Binary nanohybrids of Mn_3_O_4_/ZnO, CuO/ZnO and CuO/Mn_3_O_4_ were found to be optically responsive over a wide region of the spectrum with a maximum absorbance at around ~ 455, ~ 420 and ~ 440 nm respectively. Likewise, CuO/Mn_3_O_4_/ZnO responded across the entire range of the visible spectrum with an absorbance maximum at ~ 543 nm. Further, Tauc’s plot employed to calculate their direct band gaps showed that while pristine ZnO, Mn_3_O_4_ and CuO had band gaps measuring ~ 3.37 eV, ~ 2.14 eV and ~ 1.70 eV respectively, binary nanohybrids of Mn_3_O_4_/ZnO, CuO/ZnO and CuO/Mn_3_O_4_ had band gaps of ~ 2.60 eV, ~ 2.90 eV and ~ 2.20 eV. The ternary nanohybrid had a band gap of ~ 2.32 eV. The red shift in the absorbance edges of the nanohybrids with the ternary photocatalyst undergoing the maximum shift further confirmed the formation of integrated photocatalysts with strong interfacial interactions that ensured photocatalysis within the visible range of light. Besides, a possible intermixing of the orbitals in the valence shells of Zn, Mn and Cu species leading to the generation of conduction band at lower magnitude of energy in the nanohybrids could also be inferred^50^.

PL spectra of a semiconductor material chiefly arise due to downward electronic transition from conduction to valence band and the associated intensity can be a measure of the rate of electron–hole recombination. The nanohybrids were therefore compared with the pristine metal oxide nano-scaled samples. PL spectrum of ZnO was obtained by its excitation at 375 nm. CuO/ZnO, Mn_3_O_4_/ZnO and CuO/Mn_3_O_4_/ZnO were also excited at the same wavelength and their spectra were compared (Fig. 5b). The emission peak at ~ 406 nm might be attributed to exciton–exciton collision while emissions associated with oxygen defects occurred at ~ 467 nm and ~ 521 nm. These emissions had reduced intensities in the binary composites and had the least intensity in the final ternary nanohybrid. Likewise, CuO, CuO/ZnO, CuO/Mn_3_O_4_ and CuO/Mn_3_O_4_/ZnO were excited at ~ 449 nm and their spectra recorded (Fig. 5c). The first peak in the emission spectrum of CuO at ~ 487 nm could be ascribed to the radiative exciton annihilation while the other two peaks at ~ 500 nm and ~ 513 nm might arise from defect levels in metal oxide. Yet again, the intensity of these emissions was found reduced in the nanohybrids with the ternary composite registering the lowest intensity emissions. For comparison with Mn_3_O_4_, Mn_3_O_4_/ZnO, CuO/Mn_3_O_4_ and CuO/Mn_3_O_4_/ZnO were excited at 430 nm (Fig. 5d). The radiative recombination was intense in the pristine sample and weakened in binary nanocomposites while it was the weakest in CuO/Mn_3_O_4_/ZnO. All these data suggest that a wide separation of photogenerated electrons and holes could be achieved in the ternary nanohybrid.

## Study of the operating parameters of photodegradation of rabeprazole

### Effect of photocatalyst dosage

**Figure 6:**
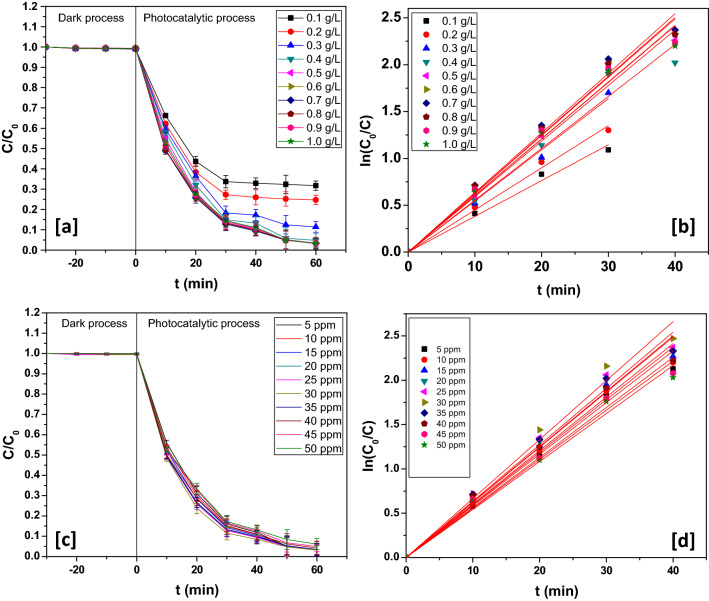
(**a**) Dynamics and (**b**) kinetics profile at different catalyst loading. (**c**) Dynamics and (**d**) kinetics profile at different rabeprazole concentration loading.

The test for optimality of photocatalyst loading was performed to avoid its redundant use. As a result, the designed photocatalyst could be used at its best with greatest efficiency. The rate of rabeprazole photodegradation in presence of different loadings of CuO/Mn_3_O_4_/ZnO is shown in Fig. 6(b). To find out the optimum amount of the designed photocatalyst required for degradation of rabeprazole, a 50 mL of 25 ppm rabeprazole solution was taken, and with an initial pH of 7, the dosage of catalyst was regulated in the range 100–1000 ppm. Maximum photodegradation yield was attained at a catalyst concentration of 700 ppm (Fig. 6a). The efficiency of photodegradation underwent a slender decline past this concentration. Although additional use of photocatalyst implies introduction of yet greater number of active sites on its surface, there occurs severe solution opacity that leads to a decline in photocatalytic activity. The velocity constant reached the greatest value (6.35 × 10^–2^ min^−1^) for 25 ppm of photocatalyst dose (Fig. 6b).

### Effect of initial rabeprazole concentration

For fitting assessment of the as to the manner the initial concentration of rabeprazole affects photodegradation, experiments were run with the optimal catalyst dosage of 700 ppm and initial pH of 7 at varying rabeprazole concentrations. Maximum degradation was achieved at 30 ppm of rabeprazole concentration (Fig. 6c). Rabeprazole concentration higher than this optimal concentration demonstrated a slight fall in the degradation efficiency. This could be ascribed to the shortening of photon path length at higher rabeprazole concentrations^51^. Furthermore, higher rabeprazole concentrations would need greater photocatalyst surface area for degradation and this could be only achieved by the addition of extra amount of photocatalyst that would invariably raise the solution opacity^52^. Therefore, optimization of rabeprazole dose enabled superlative yield of photodegradation. The velocity constant assumed the highest magnitude (6.65 × 10^–2^ min^−1^) at optimal rabeprazole concentration (Fig. 6d).

### Effect of pH

**Figure 7:**
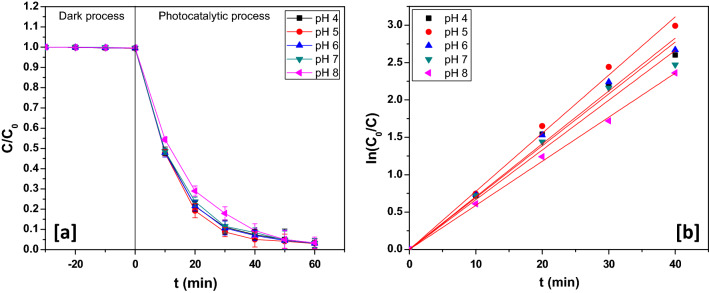
(**a**) Dynamics and (**b**) kinetics at different pH.

The effect of pH on the photocatalytic breakdown of the aqueous rabeprazole solution was studied. For this purpose, the catalyst concentration was fixed at 700 ppm and the concentration of rabeprazole was maintained at 30 ppm while the initial pH was varied from 4 to 8 during the experiments. There was a marginal increase in photodegradation yield till pH 5 and then a minor decrease was registered (Fig. 7a). The point of zero charge (pH_pzc_) of the catalyst was found to be ~ 4.8 pH. Thus with increasing the pH of the solution, large number of hydroxide ions can be accumulated over the nanocatalyst surface^53^. Hydroxide ions gathering over the photocatalyst surface at higher pH might lead to repulsion of electron-rich rabeprazole species by the catalyst. As evident from Fig. 7(b), it was at pH 5; the velocity constant acquired its maximum value (7.77 × 10^–2^ min^−1^).

### Effect of irradiation time

**Figure 8:**
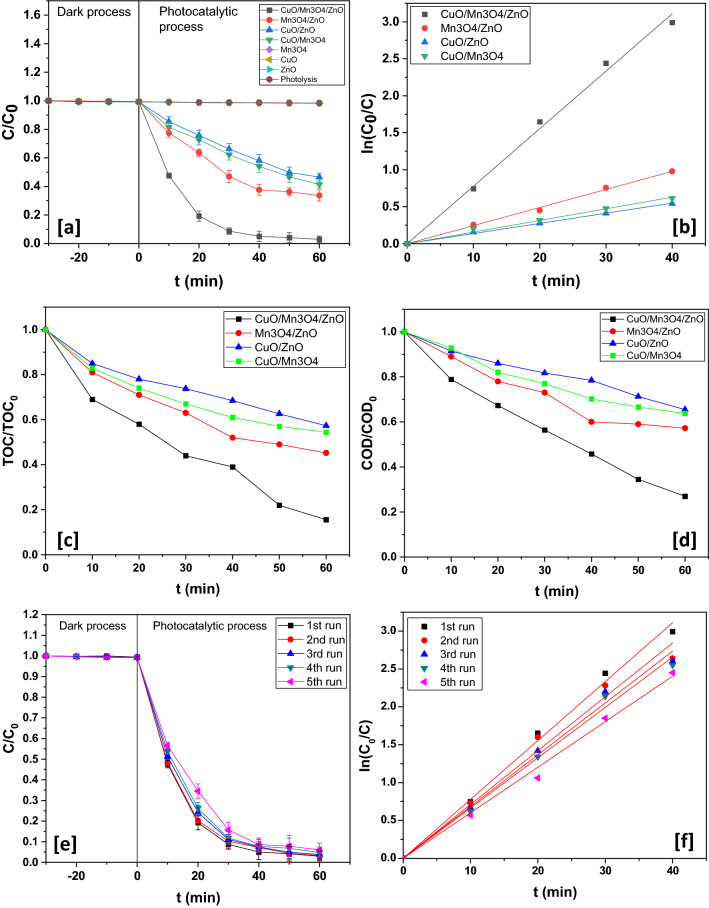
(**a**) Dynamics and (**b**) kinetics profile for different catalysts. (**c**) TOC removal profile for different catalysts. (**d**) COD reduction profile for different catalysts. (**e**) Dynamics and (**f**) kinetics profile for five consecutive runs with CuO/Mn_3_O_4_/ZnO.

The effect of irradiation time was investigated by measuring the degradation yield at different time periods under optimal operating conditions of photocatalyst dosage, initial pharmaceutical concentration and pH. The photocatalyst loading used for this purpose was 700 ppm while initial rabeprazole concentration of 30 ppm and initial pH of 5 was employed. A maximum rabeprazole decomposition of ~ 97.02 ± 1.15% was attained by 60 min past which no significant photodegradation was registered (Fig. 8a,b). The near complete exhaustion of active sites on the photocatalyst surface culminated in the apparent stoppage of the light driven degradation reaction after 60 min.

### Photocatalytic behaviour of various photocatalysts

Photolysis and pristine sample barely achieved any noticeable photodegradation activity (Fig. 8a,b). Under optimum conditions of catalyst loading, initial rabeprazole concentration and initial pH, CuO/Mn_3_O_4_/ZnO demonstrated a photodegradation efficiency of 97.02 ± 1.15% within 60 min while Mn_3_O_4_/ZnO, CuO/ZnO and CuO/Mn_3_O_4_ could attain photodegradation efficiency of 66.33 ± 1.95%, 53.43 ± 1.36% and 58.66 ± 1.28% within a period of 60 min under the same set of conditions (Fig. 8a,b). An outstanding velocity constant of 0.07773 min^−1^ was achieved during photodegradation over CuO/Mn_3_O_4_/ZnO. Furthermore, this ternary composite achieved 84.45% of TOC removal and 73.01% COD reduction. (Fig. 8c,d). Going by the photodegradation percentage, the ternary nanohybrid proved ~ 1.46 times better than Mn_3_O_4_/ZnO, ~ 1.81 times better than CuO/ZnO and ~ 1.65 times better than CuO/Mn_3_O_4_. Also, the ternary photocatalyst proved remarkably more efficient than CuSnO_3_ that took 120 min to cause ~ 87% of rabeprazole degradation at a velocity constant of 0.063 min^−1^^54^. A comparative account of the photocatalytic performance of the synthesized nanocatalyst with the previously reported catalysts has been presented in Table 2. The quantum efficiencies of the aforementioned photocatalyst systems have been calculated in Table S2 (ESI).

**Table 2:** Photodegradation chart over different catalyst systems.

Samples	Efficiency (%)	Irradiation time	k (min^−1^)	% TOC removal	% COD reduction	R^2^
CuO/Mn_3_O_4_/ZnO	97.02 ± 1.15	60	0.07773	84.45	73.01	0.99752
CuSnO_3_ Quantum Dots Ref.^54^	87	120	0.06300	–	–	0.98000
Mn_3_O_4_/ZnO	66.33 ± 1.95	60	0.02446	54.78	42.75	0.99857
CuO/ZnO	53.43 ± 1.36	60	0.01373	42.67	34.56	0.99902
CuO/Mn_3_O_4_	58.66 ± 1.28	60	0.01573	45.56	36.21	0.99575

### Reusability of the nano-scaled hybrid photocatalyst

**Figure 9:**
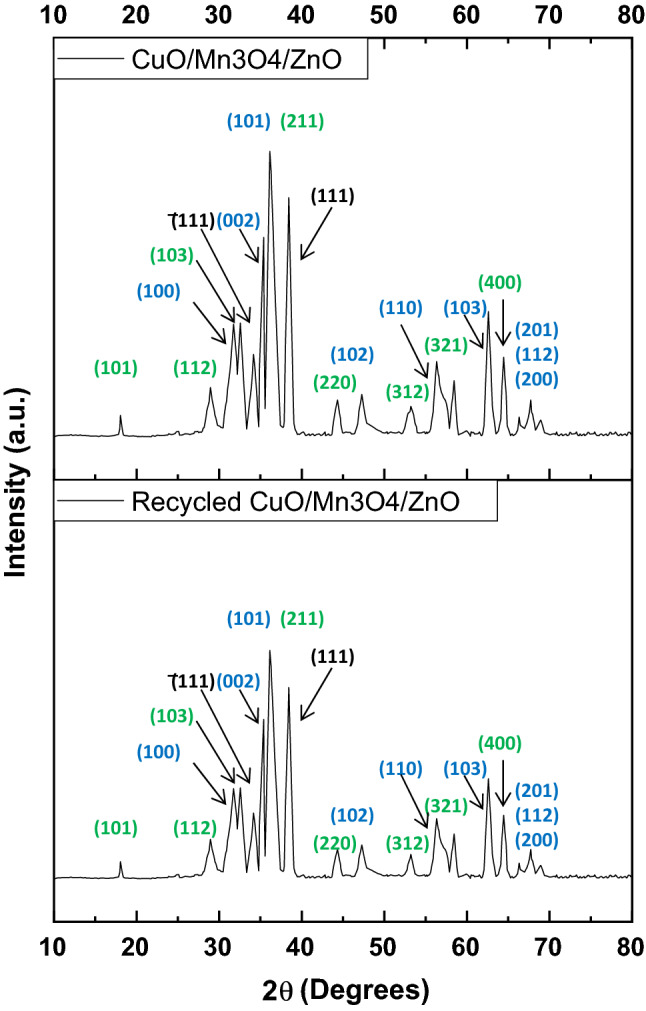
XRD patterns of CuO/Mn_3_O_4_/ZnO before and after use.

The designed hybrid photocatalyst was retrieved and could be used for five cycles in succession (Fig. 8e,f). The photocatalyst was collected by centrifugation. It was then repeatedly washed with deionized water and acetone and dried at 70 °C. The dried sample was again used for the next cycle of experiment. Degradation yield and pseudo-first order velocity constant showed a minor decrease that could be attributed to a small-scale dislodgement of the coupled photocatalyst system. Besides, XRD pattern obtained for the recycled photocatalyst showed retention of the characteristic crystallographic planes found in CuO/Mn_3_O_4_/ZnO before use (Fig. 9). This established the overall durability of the fabricated photocatalyst. The XPS of the recycled photocatalyst was also performed and as evident from Supplementary Fig. S1, the surface composition of the recycled photocatalyst and the electronic states of Zn, Mn, Cu and O remained unaltered indicating sustenance of co-existing ZnO, Mn_3_O_4_ and CuO phases after five consecutive runs of the experiment. This was additionally supported by the results of elemental analysis from EDAX spectrum (Supplementary Fig. S2) of the recycled catalyst that showed little difference from that obtained for the unused catalyst. These data revealed the stability of the ternary nanocomposite suggesting substantial interfacial interaction between the moieties and lent considerable evidence in favour of minimal leaching of metal ions during photocatalysis.

**Figure 10:**
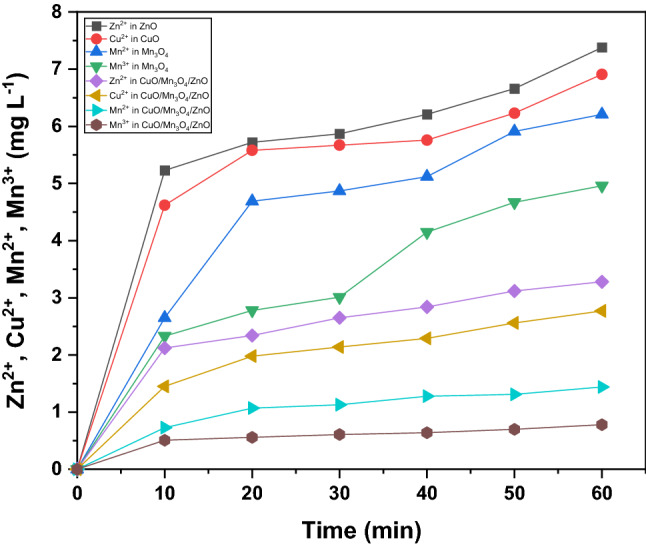
Release of various metal ions with time from different samples.

The concentrations of leached metal ions were determined during photocatalysis using pristine metal oxide components and the ternary nanocomposite (Fig. 10). During this investigation, it emerged that at total of 7.38 ppm of Zn^2+^ and 6.91 ppm of Cu^2+^ were respectively released from pristine samples of ZnO and CuO while 6.21 ppm and 4.96 ppm of Mn^2+^ and Mn^3+^ ions leached at the end of photocatalysis. This leaching dropped to 3.28 ppm, 2.77 ppm, 1.44 ppm and 0.78 ppm of Zn^2+^, Cu^2+^, Mn^2+^ and Mn^3+^ when the ternary nanocomposite was used in the experiment indicating that a greater stability was rendered to the ternary hierarchical structure. The fabrication technique so employed in the preparation of the hierarchical structure could have imparted good elemental contact and high dispersion as corroborated by TEM, XPS, EDAX and optical data leading to low aggregation of individual metal oxides within the ternary nanocomposite. Further, it has been reported that a hierarchical structure of semiconductor leads to the higher stability against aggregation^55,56^. Thus, the leaching of metal ions triggered by aggregation will be hindered by hierarchical configuration.

### Effect of scavengers

**Figure 11:**
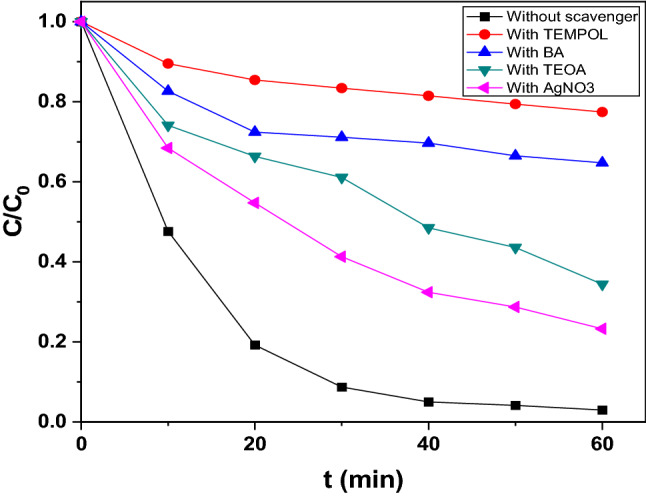
Effect of scavengers on photodegradation.

For appraisal of the roles played by reactive species such as O_2_^−·^, ^·^OH, h^+^ and e^−^, photodegradation experiments were performed in presence of quenchers. 4-hydroxy-2,2, 6,6- tetramethylpiperidinyloxy (TEMPOL), benzoic acid (BA), triethanolamine (TEOA) and silver nitrate (AgNO_3_) were used as scavengers of O_2_^−·^, ^·^OH, h^+^ and e^−^^57–60^. The photodegradation in presence of TEMPOL and BA suffered obvious retardation (Fig. 11) and a drop of efficiency from ~ 97.02 ± 1.15% with CuO/Mn_3_O_4_/ZnO nanohybrid standalone to ~ 22.55% and ~ 35.27% in presence of TEMPOL and BA respectively was noted. However, in presence of TEOA and AgNO_3_, the decline in degradation efficiency was quite low. This suggested major roles played by O_2_^−·^ and ^·^OH radicals in the photodegradation of rabeprazole.

### Proposed mechanism of photocatalysis by CuO/Mn_3_O_4_/ZnO heterojunction

**Figure 12:**
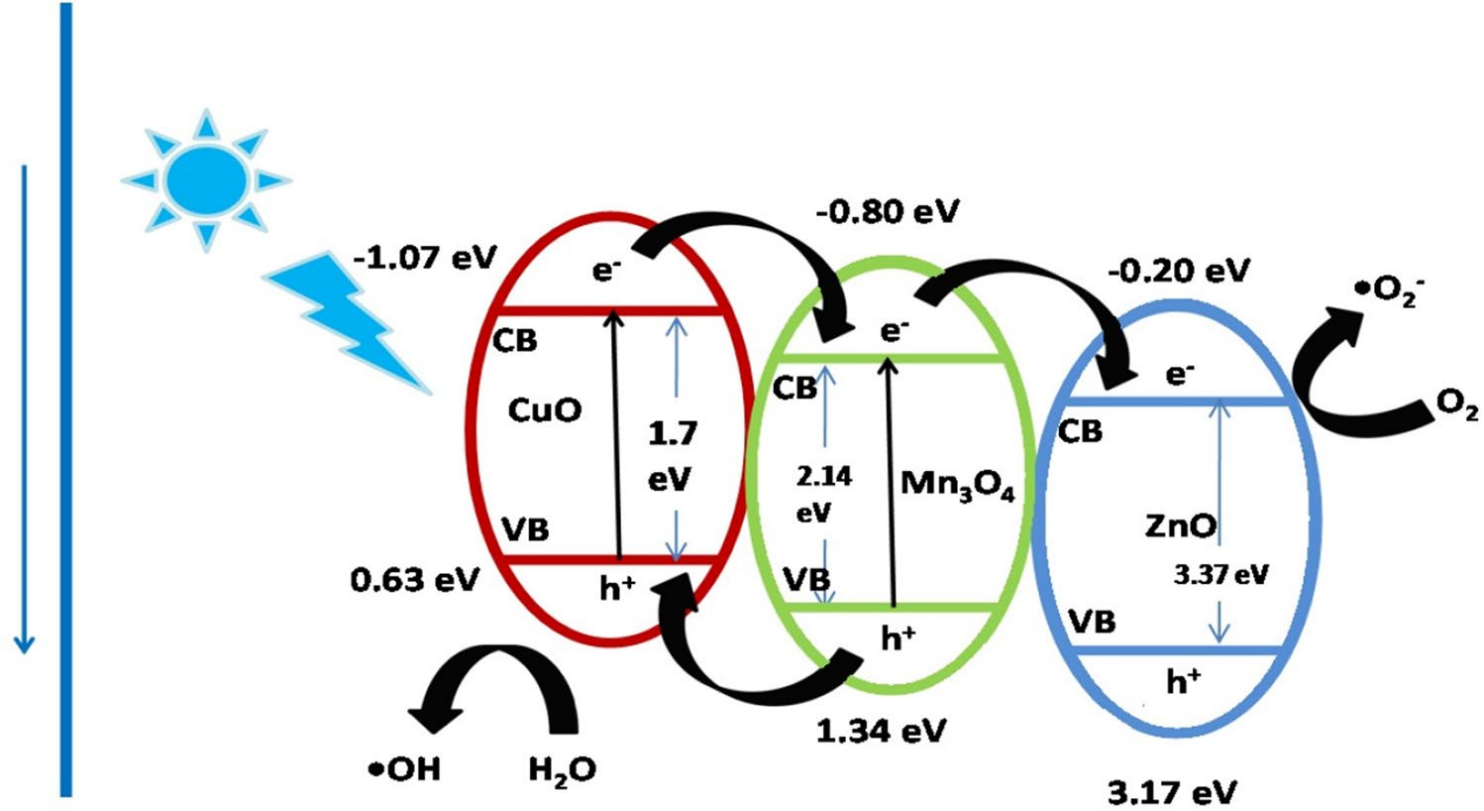
Schematic depiction of the tentative degradation mechanism by the integrated photocatalyst system.

The photocatalytic performance of the designed integrated nano-scaled photocatalyst was effectively demonstrated by its remarkable degradation efficiency and substantial TOC and COD removal ability. Besides, PL data also confirmed separation of light-induced charge carriers. Furthermore, the photocatalyst showed fairly consistent efficiency till the fifth run. These data are therefore indicative of the formation of substantial interfacial interactions between the moieties^61^. The tentative mechanism of photocatalysis by CuO/Mn_3_O_4_/ZnO is depicted in Fig. 12. The conduction band (CB) edge of CuO is stationed at -1.07 eV^36^ while that of Mn_3_O_4_ is at -0.80 eV^62^ with respect to standard hydrogen electrode (SHE). The conduction band (CB) edge of ZnO is located at -0.20 eV^63^. Invariably the conduction band (CB) of CuO has larger negative value than that of Mn_3_O_4_^64^ which in turn has larger negative value than that of ZnO. Therefore, electrons promoted from the VB of CuO to its CB as a result of absorption of photons of the visible light are transferred first to the CB of Mn_3_O_4_ and to finally accumulate in the CB of ZnO. Simultaneously, holes travel from the VB of ZnO to VB of Mn_3_O_4_ and then to the VB of CuO. These electrons at the CB of ZnO then reacted with adsorbed oxygen (O_2_) molecules to generate superoxide anion radicals (O_2_^−·^). This took place because the standard reduction potential of (O_2_/ O_2_^−·^) is 0.13 eV which is higher than the CB edge potential of ZnO^65–68^. The holes, on the other hand, reacted with water molecules (H_2_O) to introduce hydroxyl radicals (^•^OH). Generation of ^·^OH radicals might follow routes described underneath^69–72^. The synergistic effect among the moieties of the integrated photocatalyst leading to its photocatalytic abilities could be best delineated by the following equations:

3 $$ {\text{CuO}} + {\text{visible}}\,{\text{light}}\, \to \,{\text{CuO}}\left( {{\text{e}}^{ - }_{{{\text{CB}}}} } \right) + {\text{CuO}}\left( {{\text{h}}^{ + }_{{{\text{VB}}}} } \right) $$

4 $$ {\text{CuO}}\left( {{\text{e}}^{ - }_{{{\text{CB}}}} } \right) + {\text{Mn}}_{{3}} {\text{O}}_{{4}} \to {\text{CuO}} + {\text{Mn}}_{{3}} {\text{O}}_{{4}} \left( {{\text{e}}^{ - }_{{{\text{CB}}}} } \right) $$

5 $$ {\text{Mn}}_{{3}} {\text{O}}_{{4}} \left( {{\text{e}}^{ - }_{{{\text{CB}}}} } \right) \, + {\text{ ZnO }} \to {\text{ Mn}}_{{3}} {\text{O}}_{{4}} + {\text{ ZnO}}\left( {{\text{e}}^{ - }_{{{\text{CB}}}} } \right) $$

6 $$ {\text{ZnO}}\left( {{\text{e}}^{ - }_{{{\text{CB}}}} } \right) + {\text{ O}}_{{2}} \to {\text{ ZnO }} + {\text{ O}}_{{2}}^{ - \cdot } $$

7 $$ {\text{h}}^{ + }_{{{\text{VB}}}} + {\text{ H}}_{{2}} {\text{O }} \to{^ \cdot}{\text{OH }} + {\text{ H}}^{ + } $$

8 $$ {\text{h}}^{ + }_{{{\text{VB}}}} + {\text{ H}}_{{2}} {\text{O }} \to {\text{ H}}_{{2}} {\text{O}}_{{2}} + {\text{ 2H}} + $$

9 $$ {\text{H}}_{{2}} {\text{O}}_{{2}} + {\text{ e}}^{ - } \to{ \cdot }{\text{OH }} + {\text{ OH}}^{ - } $$

10 $${ \cdot }{\text{O}}_{{2}}^{ - } + {\text{ 2H}}^{ + } + {\text{ e}}^{ - } \to {\text{ H}}_{{2}} {\text{O}}_{{2}} $$

11 $$ {\text{H}}_{{2}} {\text{O}}_{{2}} + {\text{ e}}^{ - } \to{ \cdot }{\text{OH }} + {\text{ OH}}^{ - } $$

12 $$ {\text{O}}_{{2}}^{ - \cdot } /{}^{ \cdot }{\text{OH }} + {\text{ Rabeprazole}} \to {\text{Degraded}}\,{\text{products}} $$

**Figure 13:**
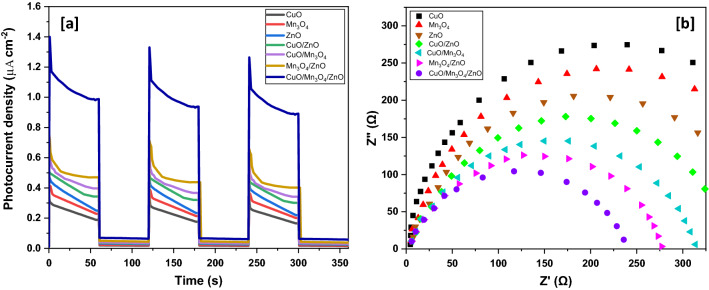
(**a**) Transient photocurrent and (**b**) EIS of various samples.

To further throw light on the aforesaid charge transfer mechanism, the separation efficiency of the photogenerated charge carriers was investigated using photocurrent response and electrochemical impedance spectra (EIS). The photocurrent densities versus the irradiation time curves of the different samples under chopped illumination are shown in Fig. 13(a). The photocurrent densities dramatically rose when the lamp was turned on and went on to sustain nearly stable values for the time the lamp was kept on. However, the photocurrents rapidly decreased to zero as soon as the lamp was turned off. The photocurrent generated by the ternary nanocomposite was much higher than the binary nanocomposites and the pristine samples. This suggested a smaller recombination and a more efficient separation of photo-generated electron–hole pairs at the interface between the moieties in the ternary nanocomposite. In other words, the remarkable improvement of the photocurrent of the ternary nanocomposite is a consequence of a longer life-span and more efficient separation of charge carriers than in individual metal oxide moieties or their binary combinations. The EIS Nyquist plots of the photocatalysts are shown in Fig. 13(b). The arc radius of the ternary nanocomposite was smaller than that of the binary nanocomposites and the pristine metal oxides suggesting a higher efficiency of charge separation and charge transfer across the interfaces of metal oxides in the ternary nanocomposite than in the binary nanocomposites and the pristine samples. These data corroborate the results of photoluminiscence investigations.

**Figure 14:**
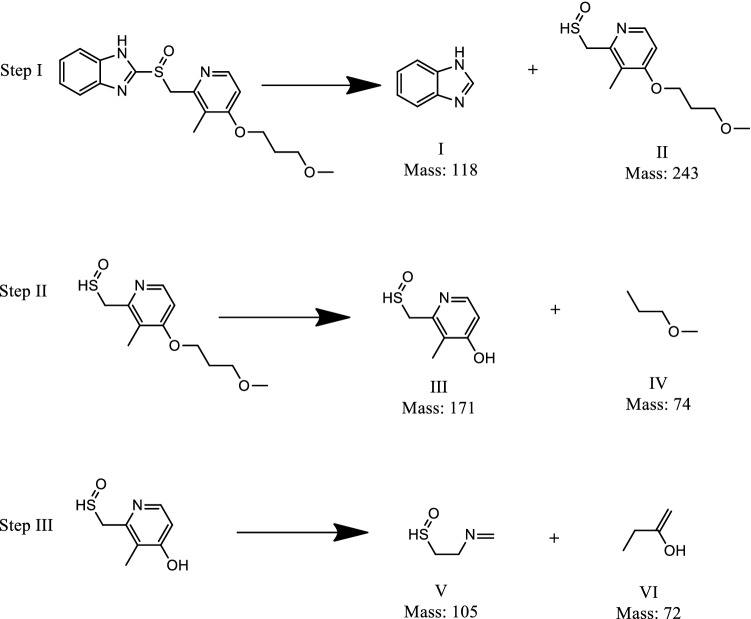
Plausible degradation route of rabeprazole.

The identification of transformation products is essential for proper determination of the route of degradation for which HRLCMS was performed at an intermediate stage of the rabeprazole photodegradation. Supplementary Figure S3 represents the liquid chromatogram resulting from HRLCMS and Supplementary Fig. S4 displays the various mass spectra emerging from this analysis, which bore out the formation of various transformation moieties that appear in the depiction of a plausible route of rabeprazole photodegradation in Fig. 14. The most probable degradation pathway involves three steps. In the first step, the parent rabeprazole molecule may undergo cleavage of C-S bond to generate two daughter fragments of mass 118 (compound I) and mass 243 (compound II). The compound II eventually may break into two parts to form compound III (mass 171) and a smaller fragment of mass 74 gets eliminated. The resultant compound III may further undergo disintegration to generate smaller degradation intermediates of mass 105 and mass 72. All these intermediates on prolonged degradation eventually get fragmented to smaller parts.

## Brief investigation on the influences of foreign species

**Figure 15:**
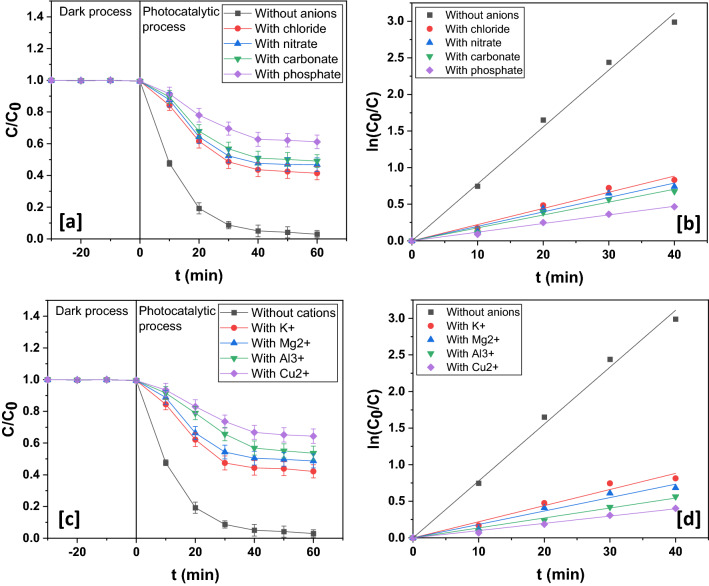
(**a**) Dynamics and (**b**) kinetics profile in presence of different anions. (**c**) Dynamics and (**d**) kinetics profile in presence of different cations.

The performance of the photocatalyst was monitored in presence of foreign species such as inorganic ions and organics. Inorganic ions of 0.05 M were added to the reaction system and the photocatalytic degradation yield was estimated in each case. There was suppression of photocatalytic activity in presence of chloride, nitrate, carbonate and phosphate (Fig. 15a,b). This was because all of them quenched hydroxyl free radicals and also displayed pronounced tendency to get adsorbed on the surface of the photocatalyst resulting in the blockage of active sites^72–76^. Usually, potassium and magnesium do not exert appreciable influences on photocatalysis. However, since their chloride salts were used, photocatalytic behaviour diminished in presence of potassium and magnesium (Fig. 15c,d)^76^. Aluminum cations are immensely adsorbed on the surface of photocatalyst thereby suppressing photodegradation yield (Fig. 15c,d)^76^. Copper (II) cations induced short-circuit by a cyclic interaction with electrons and thereafter holes and in the course impeded generation reactive species thereby exerting sizeable negative influence on photocatalysis (Fig. 15c,d)^72^.

**Figure 16:**
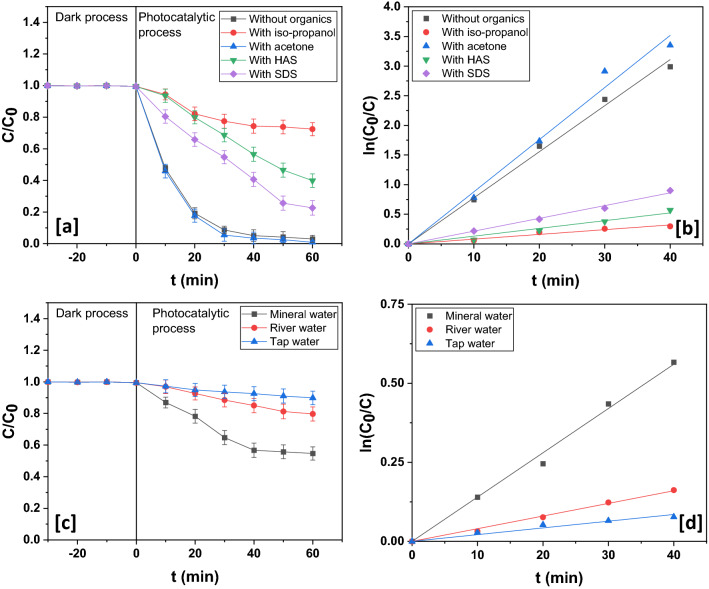
(**a**) Dynamics and (**b**) kinetics profile in presence of different organics. (**c**) Dynamics and (**d**) kinetics profile in various water samples.

In presence of organics such as isopropanol, humic acid sodium salt (HAS) and sodium dodecyl sulfate (SDS) as well, the diminishing influence was noticeable (Fig. 16a,b). Isopropanol quenches hydroxyl radicals and involves itself in preferential adsorption on the surface of photocatalyst^77^. HAS attenuates light through solution thereby slowing down photocatalysis^78^. The negative impact of SDS was chiefly due to the retarding influence of photogenerated sulfate moiety which has excellent hydroxyl scavenging properties^79^. Acetone, on the other hand, is a remarkable photosensitizer and generates hydroxyl radicals in abundance under visible light illumination (Fig. 16a,b). This explains the enhancement in photocatalytic activity upon its addition^80^.

### Influence of water samples

The grab samples of three different environmental waters were collected and their TOC and pH measured before application. The TOC of mineral water, river water and tap water was ~ 0.27 ppm, ~ 5.38 ppm and ~ 3.56 ppm while their pH was ~ 7.17, ~ 8.34 and ~ 7.75. All these water samples reduced photodegradation efficiency (Fig. 16c,d). One important reason could be light attenuation by different species present in these waters. Also, these waters, in all likelihood, abound in photocatalysis-inhibiting inorganic species and organics.

## Conclusion

In the current work, a facile fabrication of CuO/Mn_3_O_4_/ZnO nanohybrid photocatalyst was carried for an efficient photodegradation of rabeprazole, a pharmaceutical drug considered to be an emerging organic water contaminant. The structure, morphology and composition were evaluated from XRD, TEM, HRTEM, SAED, SEM and EDAX data. All these data clearly suggested the formation of the nanocomposite. The optical band gap of the nanocomposite was found to be ~ 2.32 eV and the PL, photocurrent and EIS data suggested a fair separation of light induced charge carriers and their efficient extraction as compared to binary nanocomposites and pristine metal oxides. The CuO/Mn_3_O_4_/ZnO nanostructured photocatalyst thus demonstrated excellent photocatalytic activity under visible light illumination and facilitated the decomposition of rabeprazole up to ~ 97.02 ± 1.15% of its initial concentration within a span of 60 min. The photodegradation was found to follow pseudo-first order kinetics with a velocity constant of 0.07773 min^−1^. TOC removal of ~ 84.45% could be affected during this course while the COD was reduced up to ~ 73.01%. This suggested substantial degree of mineralization and thereby the immense feasibility of photocatalytic degradation by the ternary nanohybrid of CuO/Mn_3_O_4_/ZnO. This could be possible because of the fine tuning of band edges of ZnO brought about by integration with CuO and Mn_3_O_4_. Both these moieties functioned as principal visible light harvesting units and with electronic structures compatible to each other and to ZnO, they also assisted in outright decline in the rate of recombination of photoinduced charge carriers. These charge carriers with effectively prolonged lifetime engendered reactive species that eventually led to the disintegration of the target organic water contaminant. Besides, scavenger experiment bore out the active roles played by superoxide and hydroxyl free radicals whose very generation is premised on the electron transfer mechanism so proposed thereby confirming the formation of heterojunction interfaces between the moieties of the hybrid photocatalyst. Additionally, the photocatalyst was observed to demonstrate reasonably sustained photocatalytic activity up to five runs. The lower leaching of metal ions in the ternary nanocomposite when compared to pristine samples of metal oxides further suggested considerable stability of the hybrid photocatalyst. The stability was further confirmed from XRD, XPS and EDAX investigations of the recycled catalyst that displayed retention of the crystallinity and composition of the unused catalyst. This could be attributed to stronger interfacial interactions between the moieties of the hybrid photocatalyst and high of nanoparticles of the individual metal oxides in the nanocomposite. Investigation of the photocatalyst’s performance in presence of co-existing foreign species showed that co-anions like chloride, nitrate, carbonate, and phosphate exerted a negative impact, while co-cations like potassium and magnesium ions had little bearing on photocatalysis, although aluminum and copper (II) ions offered great hindrance to photocatalytic activity. Organics save acetone too showed inhibiting influence. Acetone being a photosensitizer augmented photocatalysis through generation of additional hydroxyl radicals. Grab water samples induced retardation of photocatalysis due to suppressing actions of the various components present in them.

## Supplementary Information

Supplementary Information.

## References

[1] Ali, I., Aboul Enein, H. Y., & Kummerer, K. Analyses of drugs and pharmaceuticals in the environment. *Biophysico‐Chemical Processes of Anthropogenic Organic Compounds in Environmental Systems*, 439–462 (2011).

[2] Ghiselli G, Jardim WF (2007). Endocrine disruptors in the enviroment. Quim. Nova.

[3] John, A., Rajan, M. S., & Thomas, J. Carbon nitride-based photocatalysts for the mitigation of water pollution engendered by pharmaceutical compounds. *Environmental Science and Pollution Research*, 1–22 (2021). 10.1007/s11356-021-13528-y 33772713

[4] Dalahmeh S, Björnberg E, Elenström AK, Niwagaba CB, Komakech AJ (2020). Pharmaceutical pollution of water resources in Nakivubo wetlands and Lake Victoria, Kampala, Uganda. Sci. Tot. Environ..

[5] Barcellos DDS, Helwig K, Gervasoni R, Teedon P, Possetti GRC, Bollmann HA (2020). Priority pharmaceutical micropollutants and feasible management initiatives to control water pollution from the perspective of stakeholders in Metropolis of southern Brazil. Integr. Environ. Assess. Manag..

[6] Yu X (2020). Municipal solid waste landfills: an underestimated source of pharmaceutical and personal care products in the water environment. Environ. Sci. Technol..

[7] O'Flynn D (2021). A review of pharmaceutical occurrence and pathways in the aquatic environment in the context of a changing climate and the COVID-19 pandemic. Anal. Methods.

[8] Pallotta S, Pace F, Marelli S (2008). Rabeprazole: A second-generation proton pump inhibitor in the treatment of acid-related disease. Expert Rev. Gastroent..

[9] Gan G, Zhao P, Zhang X, Liu J, Liu J, Zhang C, Hou X (2017). Degradation of pantoprazole in aqueous solution using magnetic nanoscaled Fe_3_O4/CeO_2_ composite: Effect of system parameters and degradation pathway. J. Alloys Compd..

[10] Miner P, Orr W, Filippone J, Jokubaitis L, Sloan S (2002). Rabeprazole in nonerosive gastroesophageal reflux disease: A randomized placebo-controlled trial. Am. J. Gastroent..

[11] Bytzer P, Blum A, De Herdt D, Dubois D, Investigators T (2004). Six-month trial of on-demand rabeprazole 10 mg maintains symptom relief in patients with non-erosive reflux disease. AlimenT. Pharm. Ther..

[12] Caplan A, Fett N, Rosenbach M, Werth VP, Micheletti RG (2017). Prevention and management of glucocorticoid-induced side effects: A comprehensive review: a review of glucocorticoid pharmacology and bone health. J. Am. Acad. Dermatol..

[13] Amjad, W., Qureshi, W., Farooq, A., Sohail, U., Khatoon, S., Pervaiz, S., Narra, P., Hasan, S.M., Ali, F., Ullah, A., & Guttmann, S. Gastrointestinal Side Effects of Antiarrhythmic Medications: A Review of Current Literature. *Cureus*, *9*(9) (2017). 10.7759/cureus.1646 PMC5669531 29142794

[14] Varma, K. S., Tayade, R. J., Shah, K. J., Joshi, P. A., Shukla, A. D., & Gandhi, V. G. Photocatalytic degradation of pharmaceutical and pesticide compounds (PPCs) using doped TiO2 nanomaterials: A review. *Water-Energy Nexus *(2020).

[15] da Silva, S. W., Welter, J. B., Albornoz, L. L., Heberle, A. N. A., Ferreira, J. Z., & Bernardes, A. M. (2021). Advanced electrochemical oxidation processes in the treatment of pharmaceutical containing water and wastewater: A review. *Current Pollution Reports*, 1–14.

[16] Liqiang J, Xiaojun S, Jing S, Weimin C, Zili X, Yaoguo D, Honggang F (2003). Review of surface photovoltage spectra of nano-sized semiconductor and its applications in heterogeneous photocatalysis. Sol. Energy Mater. Sol. Cells.

[17] Khan MM, Ansari SA, Pradhan D, Han DH, Lee J, Cho MH (2014). Defect-induced band gap narrowed CeO_2_ nanostructures for visible light activities. Ind. Eng. Chem. Res..

[18] Chaniotakis N, Sofikiti N (2008). Novel semiconductor materials for the development of chemical sensors and biosensors: A review. Anal. Chim. Acta.

[19] Khan MM, Ansari SA, Lee JH, Ansari MO, Lee J, Cho MH (2014). Electrochemically active biofilm assisted synthesis of Ag@CeO_2_ nanocomposites for antimicrobial activity, photocatalysis and photoelectrodes. J. Colloid Interface Sci..

[20] Zhou H, Wen Z, Liu J, Ke J, Duan X, Wang S (2019). Z-scheme plasmonic Ag decorated WO3/Bi2WO6 hybrids for enhanced photocatalytic abatement of chlorinated-VOCs under solar light irradiation. Appl. Catal. B.

[21] Solomon RV, Lydia IS, Merlin JP, Venuvanalingam P (2012). Enhanced photocatalytic degradation of azo dyes using nano Fe_3_O_4_. J. Iran. Chem. Soc..

[22] Carré G, Hamon E, Ennahar S, Estner M, Lett MC, Horvatovich P, Gies JP, Keller V, Keller N, Andre P (2014). TiO_2_ photocatalysis damages lipids and proteins in *Escherichia coli*. Appl. Environ. Microbiol..

[23] Kabra K, Chaudhary R, Sawhney RL (2004). Treatment of hazardous organic and inorganic compounds through aqueous-phase photocatalysis: A review. Ind. Eng. Chem. Res..

[24] Wang S (2020). Bimetallic Fe/In metal-organic frameworks boosting charge transfer for enhancing pollutant degradation in wastewater. Appl. Surf. Sci..

[25] Zou X, Dong Y, Ke J, Ge H, Chen D, Sun H, Cui Y (2020). Cobalt monoxide/tungsten trioxide pn heterojunction boosting charge separation for efficient visible-light-driven gaseous toluene degradation. Chem. Eng. J..

[26] Pirhashemi M, Habibi-Yangjeh A, Pouran SR (2018). Review on the criteria anticipated for the fabrication of highly efficient ZnO-based visible-light-driven photocatalysts. J. Ind. Eng. Chem..

[27] Senasu T, Chankhanittha T, Hemavibool K, Nanan S (2021). Visible-light-responsive photocatalyst based on ZnO/CdS nanocomposite for photodegradation of reactive red azo dye and ofloxacin antibiotic. Mater. Sci. Semiconduct. Process..

[28] Adegoke KA, Iqbal M, Louis H, Bello OS (2019). Synthesis, characterization and application of CdS/ZnO nanorod heterostructure for the photodegradation of Rhodamine B dye. Mater. Sci. Energy Technol..

[29] Pung, S. Y., Chan, Y. L., Sreekantan, S., & Yeoh, F. Y. Photocatalytic activity of ZnO-MnO2 core shell nanocomposite in degradation of RhB dye. *Pigment & Resin Technology * (2016).

[30] Wang R, Hao Q, Feng J, Wang GC, Ding H, Chen D, Ni B (2019). Enhanced separation of photogenerated charge carriers and catalytic properties of ZnO–MnO_2_ composites by microwave and photothermal effect. J. Alloys Compd..

[31] Naseri A, Samadi M, Mahmoodi NM, Pourjavadi A, Mehdipour H, Moshfegh AZ (2017). Tuning composition of electrospun ZnO/CuO nanofibers: Toward controllable and efficient solar photocatalytic degradation of organic pollutants. J. Phys. Chem. C.

[32] Harish S (2017). Controlled structural and compositional characteristic of visible light active ZnO/CuO photocatalyst for the degradation of organic pollutant. Appl. Surf. Sci..

[33] Hoseinpour V, Ghaemi N (2018). Novel ZnO–MnO_2_–Cu_2_O triple nanocomposite: Facial synthesis, characterization, antibacterial activity and visible light photocatalytic performance for dyes degradation—A comparative study. Mater. Res. Express.

[34] Tedla H, Díaz I, Kebede T, Taddesse AM (2015). Synthesis, characterization and photocatalytic activity of zeolite supported ZnO/Fe_2_O_3_/MnO_2_ nanocomposites. J. Environ. Chem. Eng..

[35] Pranesh S, Nagaraju J (2020). Nano sized ZnO/MnO_2_/Gd_2_O_3_ ternary heterostructures for enhanced photocatalysis. Curr. Nanomater..

[36] Helaïli N, Bessekhouad Y, Bouguelia A, Trari M (2009). Visible light degradation of Orange II using xCu_y_O_z_/TiO2 heterojunctions. J. Hazard. Mater..

[37] Janczarek M, Kowalska E (2017). On the origin of enhanced photocatalytic activity of copper-modified titania in the oxidative reaction systems. Catalysts.

[38] Zhao J, Nan J, Zhao Z, Li N, Liu J, Cui F (2017). Energy-efficient fabrication of a novel multivalence Mn_3_O_4_–MnO_2_ heterojunction for dye degradation under visible light irradiation. Appl. Catal. B.

[39] Wang G, Huang B, Lou Z, Wang Z, Qin X, Zhang X, Dai Y (2016). Valence state heterojunction Mn_3_O_4_/MnCO_3_: Photo and thermal synergistic catalyst. Appl. Catal. B.

[40] Rangel WM, Santa RAAB, Riella HG (2020). A facile method for synthesis of nanostructured copper (II) oxide by coprecipitation. J. Market. Res..

[41] Zhang X, Sun S, Lv J, Tang L, Kong C, Song X, Yang Z (2014). Nanoparticle-aggregated CuO nanoellipsoids for high-performance non-enzymatic glucose detection. J. Mater. Chem. A.

[42] Munawar K, Mansoor MA, Basirun WJ, Misran M, Huang NM, Mazhar M (2017). Single step fabrication of CuO–MnO–2TiO_2_ composite thin films with improved photoelectrochemical response. RSC Adv..

[43] Hong ZS, Cao Y, Deng JF (2002). A convenient alcohothermal approach for low temperature synthesis of CuO nanoparticles. Mater. Lett..

[44] Xiao J, Wan L, Wang X, Kuang Q, Dong S, Xiao F, Wang S (2014). Mesoporous Mn_3_O_4_–CoO core–shell spheres wrapped by carbon nanotubes: A high performance catalyst for the oxygen reduction reaction and CO oxidation. J. Mater. Chem. A.

[45] Li N, Tian Y, Zhao J, Zhang J, Zhang J, Zuo W, Ding Y (2017). Efficient removal of chromium from water by Mn3O4@ ZnO/Mn3O4 composite under simulated sunlight irradiation: Synergy of photocatalytic reduction and adsorption. Appl. Catal. B.

[46] Al-Gaashani R, Radiman S, Daud AR, Tabet N, Al-Douri YJCI (2013). XPS and optical studies of different morphologies of ZnO nanostructures prepared by microwave methods. Ceram. Int..

[47] Wang J, Wang Z, Huang B, Ma Y, Liu Y, Qin X, Zhang X, Dai Y (2012). Oxygen vacancy induced band-gap narrowing and enhanced visible light photocatalytic activity of ZnO. ACS Appl. Mater. Interfaces.

[48] Wang F, Yang H, Zhang Y (2018). Enhanced photocatalytic performance of CuBi_2_O_4_ particles decorated with Ag nanowires. Mater. Sci. Semicond. Process..

[49] Tang JY, Guo RT, Zhou WG, Huang CY, Pan WG (2018). Ball-flower like NiO/g-C_3_N_4_ heterojunction for efficient visible light photocatalytic CO_2_ reduction. Appl. Catal. B.

[50] Taufik A, Susanto IK, Saleh R (2018). Magnetically separable and reusable Fe_3_O_4_/ZnO/nanographene platelets photocatalyst for the removal of dye. J. Phys. Conf. Ser..

[51] Houas A, Lachheb H, Ksibi M, Elaloui E, Guillard C, Herrmann JM (2001). Photocatalytic degradation pathway of methylene blue in water. Appl. Catal. B.

[52] Georgaki I, Vasilaki E, Katsarakis N (2014). A study on the degradation of carbamazepine and ibuprofen by TiO_2_ & ZnO photocatalysis upon UV/visible-light irradiation. Am. J. Anal. Chem..

[53] Mohanta D, Ahmaruzzaman M (2020). A novel Au-SnO2-rGO ternary nanoheterojunction catalyst for UV-LED induced photocatalytic degradation of clothianidin: Identification of reactive intermediates, degradation pathway and in-depth mechanistic insight. J. Hazard. Mater..

[54] Mohanta D, Raha S, Gupta SV, Ahmaruzzaman M (2019). Bioinspired green synthesis of engineered CuSnO_3_ quantum dots: An effective material for superior photocatalytic degradation of Rabeprazole. Mater. Lett..

[55] Li X, Yu J, Jaroniec M (2016). Hierarchical photocatalysts. Chem. Soc. Rev..

[56] Wang X, Cai W, Lin Y, Wang G, Liang C (2010). Mass production of micro/nanostructured porous ZnO plates and their strong structurally enhanced and selective adsorption performance for environmental remediation. J. Mater. Chem..

[57] Kumar PS, Selvakumar M, Babu SG, Induja S, Karuthapandian S (2017). CuO/ZnO nanorods: An affordable efficient pn heterojunction and morphology dependent photocatalytic activity against organic contaminants. J. Alloys Compd..

[58] Kumar A, Kumar A, Sharma G, Naushad M, Veses RC, Ghfar AA, Stadler FJ, Khan MR (2017). Solar-driven photodegradation of 17-β-estradiol and ciprofloxacin from waste water and CO_2_ conversion using sustainable coal-char/polymeric-g-C_3_N_4_/RGO metal-free nano-hybrids. New J. Chem..

[59] Sharma G, Kumar A, Sharma S, Ala'a H, Naushad M, Ghfar AA, Ahamad T, Stadler FJ (2019). Fabrication and characterization of novel Fe0@ Guar gum-crosslinked-soya lecithin nanocomposite hydrogel for photocatalytic degradation of methyl violet dye. Sep. Purif. Technol..

[60] Jiang L, Liu H, Yuan J, Shangguan W (2010). Hydrothermal preparation and photocatalytic water splitting properties of ZrW_2_O_8_. J. Wuhan Univ. Technol. Mater. Sci. Ed..

[61] Ahmed SN, Haider W (2018). Heterogeneous photocatalysis and its potential applications in water and wastewater treatment: a review. Nanotechnology.

[62] Wu Y, Chu D, Yang P, Du Y, Lu C (2015). Ternary mesoporous WO_3_/Mn_3_O_4_/N-doped graphene nanocomposite for enhanced photocatalysis under visible light irradiation. Catal. Sci. Technol..

[63] Chidhambaram N, Ravichandran K (2017). Fabrication of ZnO/g-C_3_N_4_ nanocomposites for enhanced visible light driven photocatalytic activity. Mater. Res. Express.

[64] Yang Y, Xu D, Wu Q, Diao P (2016). Cu_2_O/CuO bilayered composite as a high-efficiency photocathode for photoelectrochemical hydrogen evolution reaction. Sci. Rep..

[65] Paul DR, Gautam S, Panchal P, Nehra SP, Choudhary P, Sharma A (2020). ZnO-modified g-C_3_N_4_: A potential photocatalyst for environmental application. ACS Omega.

[66] Lin H, Ye H, Li X, Cao J, Chen S (2014). Facile anion-exchange synthesis of BiOI/BiOBr composite with enhanced photoelectrochemical and photocatalytic properties. Ceram. Int..

[67] Yosefi L, Haghighi M (2018). Fabrication of nanostructured flowerlike p-BiOI/p-NiO heterostructure and its efficient photocatalytic performance in water treatment under visible-light irradiation. Appl. Catal. B.

[68] Li H, Jin Z, Sun H, Sun L, Li Q, Zhao X, Jia CJ, Fan W (2014). Facile fabrication of p-BiOI/n-Zn_2_SnO_4_ heterostructures with highly enhanced visible light photocatalytic performances. Mater. Res. Bull..

[69] He Y, Cai J, Li T, Wu Y, Lin H, Zhao L, Luo M (2013). Efficient degradation of RhB over GdVO_4_/g-C_3_N_4_ composites under visible-light irradiation. Chem. Eng. J..

[70] Malwal D, Gopinath P (2015). Fabrication and characterization of poly (ethylene oxide) templated nickel oxide nanofibers for dye degradation. Environ. Sci. Nano.

[71] Hsiao PH, Li TC, Chen CY (2019). ZnO/Cu_2_O/Si nanowire arrays as ternary heterostructure-based photocatalysts with enhanced photodegradation performances. Nanoscale Res. Lett..

[72] Chen HY, Zahraa O, Bouchy M (1997). Inhibition of the adsorption and photocatalytic degradation of an organic contaminant in an aqueous suspension of TiO_2_ by inorganic ions. J. Photochem. Photobiol., A.

[73] Chládková B, Evgenidou E, Kvítek L, Panáček A, Zbořil R, Kovář P, Lambropoulou D (2015). Adsorption and photocatalysis of nanocrystalline TiO_2_ particles for reactive red 195 removal: Effect of humic acids, anions and scavengers. Environ. Sci. Pollut. Res..

[74] Liu Y, He X, Duan X, Fu Y, Dionysiou DD (2015). Photochemical degradation of oxytetracycline: Influence of pH and role of carbonate radical. Chem. Eng. J..

[75] Hu C, Jimmy CY, Hao Z, Wong PK (2003). Effects of acidity and inorganic ions on the photocatalytic degradation of different azo dyes. Appl. Catal. B.

[76] Wang C, Zhu L, Wei M, Chen P, Shan G (2012). Photolytic reaction mechanism and impacts of coexisting substances on photodegradation of bisphenol A by Bi2WO6 in water. Water Res..

[77] Chen Y, Yang S, Wang K, Lou L (2005). Role of primary active species and TiO_2_ surface characteristic in UV-illuminated photodegradation of Acid Orange 7. J. Photochem. Photobiol. A.

[78] Li S, Hu J (2016). Photolytic and photocatalytic degradation of tetracycline: Effect of humic acid on degradation kinetics and mechanisms. J. Hazard. Mater..

[79] Hidaka H, Nohara K, Ooishi K, Zhao J, Serpone N, Pelizzetti E (1994). Photodegradation of surfactants. XV: Formation of SO42− ions in the photooxidation of sulfur-containing surfactants. Chemosphere.

[80] Haroune L, Salaun M, Ménard A, Legault CY, Bellenger JP (2014). Photocatalytic degradation of carbamazepine and three derivatives using TiO_2_ and ZnO: Effect of pH, ionic strength, and natural organic matter. Sci. Total Environ..

