# Supplementary material for: Novel CuO/Mn3O4/ZnO nanocomposite with superior photocatalytic activity for removal of Rabeprazole from water
Source: Sci Rep. 2021 Jul 26;11:15187. doi: 10.1038/s41598-021-94066-y (PMC8313665; doi:10.1038/s41598-021-94066-y)
Supplement: Supplementary file 1 — Supplementary Information. [file 41598_2021_94066_MOESM1_ESM.doc]

Electronic supplementary information

**Novel CuO/Mn_3_O_4_/ZnO nanocomposite with superior photocatalytic activity for removal of Rabeprazole from water**

Sauvik Raha, Dipyaman Mohanta, and Md. Ahmaruzzaman^*^

Department of Chemistry, National Institute of Technology Silchar - 788010, Assam, India

^*^Email: [mda2002@gmail.com](mailto:mda2002@gmail.com)

Figure S1. (a) XPS survey spectrum of recycled catalyst (CuO/Mn_3_O_4_/ZnO) and HR-XPS spectra of (b) Cu, (c) Mn, (d) Zn and (e) O in the recycled catalyst.

Figure S2. EDAX spectrum of recycled catalyst (CuO/Mn_3_O_4_/ZnO).

Figure S3. Liquid Chromatogram for the separation of degradation products of rabeprazole.

Figure S4. Mass spectra identifying various degradation products of rabeprazole.

Figure S1

Figure S2


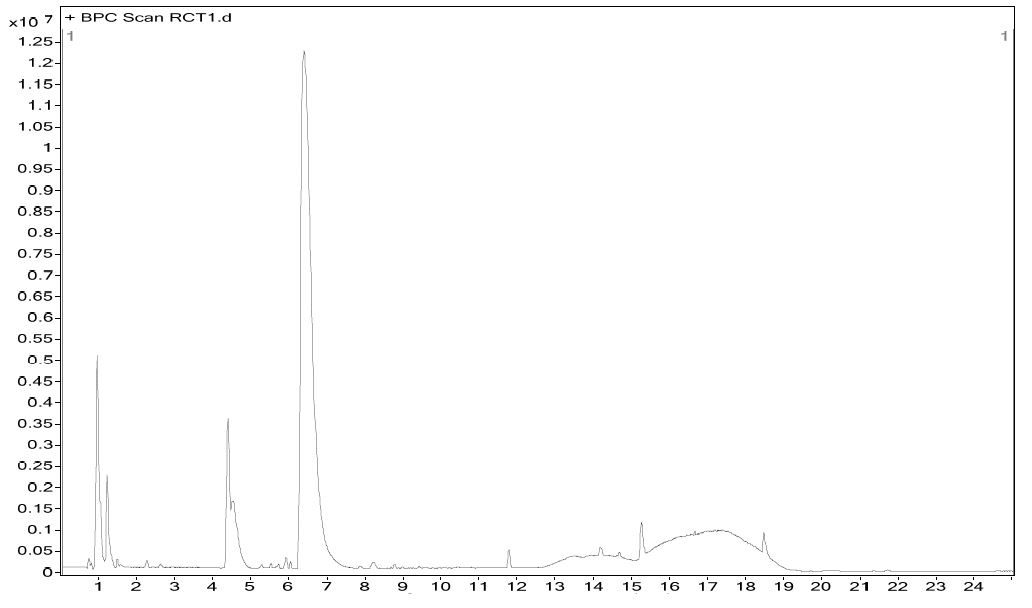


Figure S3

Figure S4

Table S1. Elemental composition of the recycled catalyst (CuO/Mn_3_O_4_/ZnO) from EDAX spectrum

| Element | Line Type | Weight% | Atomic% |
| --- | --- | --- | --- |
| O | K-series | 24.76 | 54.49 |
| Cu | K-series | 16.30 | 9.10 |
| Mn | K-series | 42.30 | 27.37 |
| Zn | K-series | 16.64 | 9.02 |
| Total |  | 100 | 100 |

Table S2. Calculation of apparent quantum efficiencies.

| Details | Unit | CuO/Mn_3_O_4_/ZnO | Mn_3_O_4_/ZnO | CuO/ZnO | CuO/Mn_3_O_4_ |
| --- | --- | --- | --- | --- | --- |
| **Step 1: Calculation of number of degraded rabeprazole molecules** | | | | | |
| Rabeprazole solution | L | 0.05 | 0.05 | 0.05 | 0.05 |
| Rabeprazole concentration | ppm (mgL^-1^) | 30 | 30 | 30 | 30 |
| Molar mass of rabeprazole | g mol^-1^ | 359.44 | 359.44 | 359.44 | 359.44 |
| Moles of rabeprazole | mol | 4.17 × 10^-3^ | 4.17 × 10^-3^ | 4.17 × 10^-3^ | 4.17 × 10^-3^ |
| Molecules of rabeprazole | molecule | 0.025 × 10^20^ | 0.025 × 10^20^ | 0.025 × 10^20^ | 0.025 × 10^20^ |
| Rabeprazole degradation | % | 97.02 | 66.33 | 53.43 | 58.66 |
| No. of degraded rabeprazole molecules | molecule | 0.024255× 10^20^ | 0.0165825 × 10^20^ | 0.0133575 × 10^20^ | 0.014665 × 10^20^ |
| **Step 2: Calculation of the total number of incident photons** | | | | | |
| h (Planck’s constant) | J s | 6.6 × 10^-34^ | 6.6 × 10^-34^ | 6.6 × 10^-34^ | 6.6 × 10^-34^ |
| c (speed of light) | m s^-1^ | 3 × 10^8^ | 3 × 10^8^ | 3 × 10^8^ | 3 × 10^8^ |
| λ (wavelength of light) | nm | 539 | 539 | 539 | 539 |
| LED power | Wm^-2^ | 48.25 | 48.25 | 48.25 | 48.25 |
| Surface area of reactor | m^2^ | 1.96 × 10^-3^ | 1.96 × 10^-3^ | 1.96 × 10^-3^ | 1.96 × 10^-3^ |
| Total number of photons | photon | 9.26 × 10^26^ | 9.26 × 10^26^ | 9.26 × 10^26^ | 9.26 × 10^26^ |
| Quantum efficiency | Rabeprazole molecule photon^-1^ | 2.62 × 10^-9^ | 1.79 × 10^-9^ | 1.44 × 10^-9^ | 1.58 × 10^-9^ |

**Calculation of quantum efficiency**

The quantum efficiency (QE) has been calculated using the following formula

**QE = 1240 x (R/λ)**

where '**R**' is Photoresponsivity and λ in the wavelength of incident light
